# Management disclosure of risk factors and COVID-19

**DOI:** 10.1186/s40854-023-00459-5

**Published:** 2023-02-17

**Authors:** Tim Loughran, Bill McDonald

**Affiliations:** University of Notre Dame, Notre Dame, USA

**Keywords:** Pandemic, COVID-19, Form 10-K, Risk factors

## Abstract

Public companies in the United States are required to file annual reports (i.e., Form 10-K) and disclose, among other things, the risk factors that may harm their stock price. The risk of a pandemic was well-known before the recent crisis, and we now know that the initial impact on many shareholders was significant and negative. To what extent did managers forewarn their shareholders about this valuation risk? We examine all 10-K filings from 2018, before any knowledge of the current pandemic, and find that less than 21% of them contain any reference to pandemic-related terms. Given the management’s presumed in-depth knowledge of their business and the general awareness that pandemics have been identified as a significant global risk for at least the past decade, this number should have been higher. We find an unexpectedly positive correlation (0.137) between the use of pandemic-related words in annual reports and realized stock returns during the actual pandemic at the industry level. Some industries most severely impacted by COVID-19 barely mentioned pandemic risk in their financial disclosures to shareholders, indicating that managers were ineffective in highlighting their exposure to pandemic risks to investors.

Hindsight has more insight than foresight.

## Introduction

The Securities and Exchange Commission (SEC) imposes reporting obligations on all firms with publicly traded securities. These reports are filed electronically and made available to the public on the SEC’s Electronic Data Gathering, Analysis, and Retrieval (EDGAR) website (https://www.sec.gov/edgar.shtml). The most comprehensive, and arguably most important, of these filings is the annual Form 10-K.^1^ Form 10-K addresses 15 SEC-specified “Items”, such as “Risk Factors”, “Management’s Discussion and Analysis”, and “Financial Statements”. Although Form 10-K is sometimes referred to as the “annual report”, it differs from the “annual report to shareholders”, which is typically made available to shareholders on the investor relations page of a firm.

The 10-K—specifically Item 1A of the filing—should inform investors about the firm’s operational, legal, health, and environmental risks. Clearly, the COVID-19 pandemic had a significant negative effect on numerous US firms. For example, Fahlenbrach et al. (2021) examined the importance of financial flexibility during the COVID shock and documented that the average US firm experienced a − 37.8% decline in value from February 3, 2020, to March 23, 2020. Meanwhile, Altig et al. (2020) demonstrated that due to COVID-19 in the United States, the uncertainty proxied by implied stock market volatility, newspaper-based policy uncertainty, and even Twitter chatter about economic uncertainty increased dramatically. Baker et al. (2020) argued that strong US government restrictions on commercial activity and voluntary social distancing had a much greater impact on the stock market than previous pandemics.

Using a large sample of firms in 61 economies, Ding et al. (2021) found that certain firm characteristics, such as more cash, less debt, and less entrenched executives, resulted in a milder stock price decline due to COVID. Moreover, Ramelli and Wagner (2020) demonstrated that firms with greater exposure to trade with China underperformed during the COVID-19 crisis.^2^ In a textual analysis paper, Larcker et al. (2020) focused on the use of COVID-19 terminology (e.g., *COVID, pandemic, epidemic, face mask, hand sanitizer, social distancing,* and *lockdown*) in SEC filings between January 1 and May 29, 2020. They documented the speed with which firms disclosed COVID information to investors *after* the pandemic had begun. One of their intriguing findings is that National Beverage (the manufacturer of La Croix) made no mention of COVID in any of their financial disclosures through May 29, 2020, even as the virus was destroying the global economy. In contrast to Larcker et al. (2020) and prior research, we focus on firm disclosure to investors *before* the pandemic. Although the literature on the financial impacts of COVID is expanding rapidly, we are unaware of any that examines the correlation between managers’ foresight in acknowledging this risk when discussing the firm’s risk factors.

In his Bloomberg blog, Matt Levine somewhat jokingly—with emphasis on “somewhat”—argues that all crimes or fraudulent activities associated with companies can be prosecuted as securities fraud. If a CEO is involved in an inappropriate liaison with an employee, it may be difficult to successfully prosecute in criminal court. However, if an executive of the firm is aware of this information and it is not made public, management has violated their obligation to disclose value-relevant information. The key point here, which has also been made in the academic literature (see Healy and Palepu 2001; Nagar et al. 2003), is that managers have a strong incentive to address the SEC’s required disclosures in a transparent and relatively exhaustive manner.

The core theory of disclosure evolves from Akerloff’s (1970) result of market failure when product quality is uncertain in the presence of information asymmetry. Grossman and Hart (1980) and Milgrom (1981) showed that the conclusion of market failure can be avoided through disclosure and that, in equilibrium, there is an incentive for full disclosure. A branch of this literature focuses on disclosure in the context of managers signaling firm value. Full disclosure results become more nuanced in this literature as the assumptions of costless disclosure, certain information, and timing dynamics are considered.^3^ Much of the empirical research on this topic focuses on management’s disclosure of earnings-related information. Although this literature provides a framework for understanding whether we expect managers to disclose COVID-related information, the threshold for disclosure attributable to disclosure costs and other extensions do not provide a conclusive answer.

Note that the context for disclosure in our application differs from the typical context in which earnings-specific information is communicated. In our case, a company is required to describe all the material risks it may face. This disclosure will likely be repeated each year in the 10-K filing, barring any significant changes to the firm’s operating environment. This is more of an official recognition of risks that firms are likely already aware of, but which are not on the immediate event horizon and may be overlooked. We would argue that the simple concept of mandated disclosure, with incentives directly tied to shareholder lawsuits, would suggest that management should list broad macro risks fairly exhaustively. Thus, we will assume that managers who are well-informed and aware of potential macro risks have every incentive to disclose them.

Typical 10-K filings are lengthy, and one might surmise that few people, other than Warren Buffet, read them in their entirety. For instance, Li (2008) reported that the average 10-K between 1994 and 2004 contained over 31,000 words, whereas Loughran and McDonald (2017) documented the paucity of investors and analysts who directly downloaded annual reports from EDGAR. In a two-day window surrounding the Form 10-K filing on EDGAR, Loughran and McDonald (2017) found that investors access annual reports of publicly traded companies 28.4 times on average. Meanwhile, Cohen et al. (2020) examined the variation in annual and quarterly filings from year to year. They reported that firms with substantial changes to their disclosures underperform those with only minor modifications. The three authors argue that investors are inattentive to these simple changes in financial disclosures because there is no announcement effect on the filing date. Thus, managers can potentially protect themselves from legal liability, as it is unlikely that the majority of individuals will notice any pertinent information they may reveal in the periodic filing.

In fact, most large companies provide a comprehensive discussion of risks. The typical discussion of risk factors is gloomy, and it is difficult to imagine many investors immediately purchasing stock after reading this pessimistic information. For example, Sears Hometown & Outlet Stores devotes 11,844 words across 19 pages to describing their risks in their 2018 10-K filing. Meanwhile, Southwest Airlines discloses their risks in 4538 words across 9 pages. Southwest acknowledges that events with a low probability, such as “fires, floods, earthquakes, tornadoes, hurricanes, power loss, computer and telecommunications failures, acts of war or terrorism, computer viruses, and security breaches…,” warrant disclosure. Similarly, Bed, Bath & Beyond, whose stock was significantly lower in 2020, lists “housing markets, recession, inflation, deflation, consumer credit availability, consumer debt levels, fuel and energy costs, interest rates, tax rates and policy, unemployment trends, the impact of natural disasters, civil disturbances and terrorist activities, foreign currency exchange rate fluctuation…” None of the pandemic-related terms we will discuss later are mentioned in any of these disclosures.

Companies, particularly those in sensitive industries, should have anticipated pandemic risk. Long before the pandemic, the World Health Organization, the Global Preparedness Monitoring Board, Bill Gates, and others identified this risk as nontrivial and consequential. Furthermore, articles from credible sources in the popular press highlighted the likelihood of a global crisis prior to its occurrence.^4^ Given the stock market’s reaction to the COVID-19 pandemic, it is abundantly clear that this risk is relevant to investors. The 1918 influenza pandemic and more recent outbreaks of SARS, MERS, H1N1, and Ebola are clear examples of pandemic risk potential. For instance, the Centers for Disease Control and Prevention (CDC) estimates that the 2009 H1N1 Pandemic resulted in 12,469 deaths in the United States, a number quickly surpassed by COVID-19.^5^

We contribute to the recently developed literature on the economic aspects of the COVID pandemic by investigating whether managers discussed the potential impact of a pandemic on their business prior to the outbreak. The COVID pandemic presents a unique circumstance in which an event with a relatively low probability could substantially impact a firm’s economic well-being. In retrospect, given the scientific community’s pre-COVID assessment that we were likely to experience a significant pandemic event, firms should have incorporated a discussion of this risk in their shareholder disclosures, particularly those firms that were more susceptible to such an event. We apply natural language processing techniques to 10-K filings to determine what percentage of firms addressed this risk.^6^

We found that in 2018, less than 21% of firms included a pandemic-related word or phrase in Item 1A: Risk Factors of their 10-K. The most frequently occurring pandemic-related words are *pandemic, epidemic, health concerns, contagious disease, Avian Flu,* and *infectious* disease. Once COVID-19 began to devastate the financial markets in February–March 2020, one should expect a strong negative correlation between an industry’s propensity to warn of pandemic risks and their actual stock returns. In other words, industries with the greatest potential for economic disruptions during any pandemic should be the most forthright in alerting their shareholders of this risk. Once the pandemic struck, the stock performance of these high-risk industries would be negatively affected by the crisis. Therefore, there should be a negative relationship between pre-event pandemic-related word usage and actual stock returns during the pandemic.

Interestingly, we do not find this relationship. We observe a correlation of 0.137 between the percentage of firms in a Fama and French (1997) industry that report at least one pandemic-related word and realized stock returns between February 3, 2020, and March 23, 2020. In hindsight, managers failed to adequately warn shareholders about the pandemic risk associated with their industry.

## Data

We download all Form 10-K filings for the 2018 calendar year from the SEC’s EDGAR website. From this group, we only include filings for which we can identify “Item 1A. Risk Factors” programmatically, as this is the section of the filing that we expect to be most relevant to our analysis. Note that the SEC does not require smaller reporting companies to include this section. The Fama and French (1997) industry classification scheme classifies firms into 48 industries. Companies in the Healthcare, Medical Equipment, Pharmaceutical Products, and Wholesale Drugs industries are excluded from the analysis because they are likely to use pandemic-related terms as part of their operations. This process yields a final sample size of 2427 filings. Refer to the Appendix for additional data and parsing information.

The year 2018 provides a relatively current sample and free of any form of forward-looking bias. In other words, even though companies should have been aware of pandemics as a risk factor in 2018, none were aware of the events that transpired in 2020.

From the Form 10-K filings that have been downloaded, we can programmatically scan each document and count the number of occurrences of our targeted words. We utilize the following terms as targets: “*avian flu*”, “*contagious disease*”, “*contagious illness*”, “*corona virus*”, “*Ebola*”, “*epidemic*”, “*fear of contagion*”, “*H1N1*”, “*health concerns*”, “*infectious disease*”, “*infectious outbreak*”, “*influenza virus*”, “*localized illnesses*”, “*MERS*”, “*outbreak of disease*”, “*pandemic*”, “*SARS*”, and “*swine flu*”.^7^

## Empirical results

Summary statistics for each of the keywords are presented in Table 1. Only 509, or less than 21%, of the 2427 firms in our sample use any of the terms listed above at least once in their filings. The term *pandemic* appears the most frequently among our target words, with 436 occurrences in 12.6% of the filings. *Epidemic* comes in at a distant second with 238 total occurrences and appearances in 6.6% of documents. *Coronavirus* and *infectious outbreak* are not mentioned in any of these filings’ risk factor sections. The term *localized illnesses* appears twice in Chipotle’s 10-K filing but nowhere else. Considering only the 497 firms that use at least one of the terms, the average number of occurrences (across all terms) is slightly more than two.

**Table 1:** Summary statistics for targeted words and phrases for 2427 firm 10-Ks filed in 2018-sorted by total counts

Word/phrase	Mean number of occurrences	Standard deviation	Total count across all filings	Maximum count in one 10-K filing	% of Filings with at least one occurrence
*Pandemic*	0.180	0.593	436	9	12.6
*Epidemic*	0.098	0.462	238	9	6.6
*Health Concerns*	0.058	0.308	140	6	4.4
*Contagious Disease*	0.026	0.211	64	3	1.9
*Avian Flu*	0.018	0.184	43	5	1.3
*Infectious Disease*	0.016	0.150	39	3	1.3
*H1N1*	0.014	0.135	35	2	1.2
*Outbreak of Disease*	0.011	0.133	27	3	0.87
*SARS*	0.009	0.107	22	3	0.82
*Swine Flu*	0.008	0.118	20	3	0.62
*Ebola*	0.005	0.078	13	2	0.49
*MERS*	0.004	0.130	9	6	0.12
*Contagious Illness*	0.003	0.076	8	3	0.25
*Fear of Contagion*	0.001	0.035	3	1	0.12
*Influenza Virus*	0.001	0.035	3	1	0.12
*Localized Illnesses*	0.001	0.041	2	2	0.04
*Coronavirus*	0	0	0	0	0
*Infectious Outbreak*	0	0	0	0	0
Sum of All Occurrences	0.454	1.268	1102	18	20.97

Based on the total number of terms used in a single filing, the top four firms, with counts ranging from 10 to 17, are all firms whose predominant market is in Asia (i.e., Sino Agro Food, Yum China Holding, Air Lease Corp, and Senmiao Technology Ltd.). Apparently, the geographic concentration of previous SARS and MERS outbreaks has made these companies more aware of the potential risks associated with health issues. CNO Financial Group, a firm of health insurance and general insurance policies, is the firm with the highest usage of the term *pandemic*. In their risk factors discussion, they include the following subsection: “The occurrence of natural or man-made disasters or a pandemic could adversely affect our financial condition and results of operations.” The section contains two paragraphs, but no specifics are provided beyond the acknowledgment of a potential pandemic.

Not surprisingly, the top three industries among the 48 Fama–French (1997) industry categories with at least one reference to a pandemic-related word or phrase are Restaurants, Hotels, and Motels (75%), Food (68%), and Candy and Soda (50%). Four industries, namely, Textiles, Shipbuilding and Railroad Equipment, Gold, and Coal, had zero occurrences across all filings within the industry. Only about 30% of the filings in the retail sector, where COVID-19 has negatively impacted many firms, include these terms.

If managers could perfectly anticipate the impact of a pandemic event, we would expect their acknowledgment of the risk to mirror the corresponding stock return once the event occurs. In particular, there should be a strong negative correlation between an industry’s propensity to discuss pandemic risks and its stock performance during such an event. Using Center for Research in Security Prices stock return data, we calculate the average return over the COVID interval, which Fahlenbrach et al. (2021) defined as the trading days from 2/3/2020 to 3/23/2020, for each of the Fama–French industries.

**Fig. 1:**
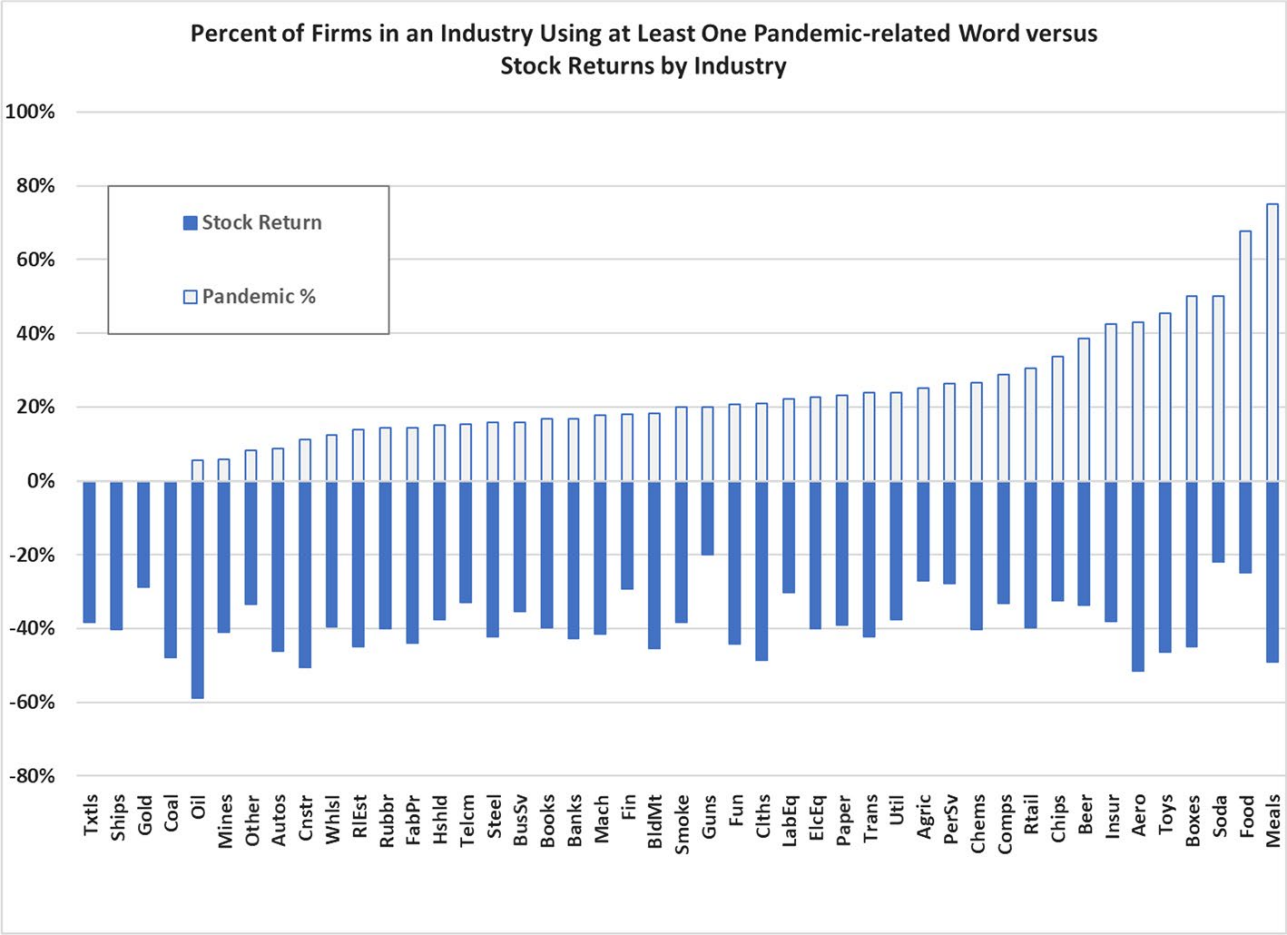
Percent of firms in an industry using at least one pandemic-related word versus stock returns by industry

Figure 1 depicts the stock returns for each industry and the proportion of firms within each industry that use at least one pandemic-related term. In a perfect information environment, we would expect the darkly shaded stock returns to correspond with the percentage of firms employing pandemic terms. The graph demonstrates little correlation between the acknowledged risk and the actual risk. The correlation between the two series, which we expect to be negative, is 0.137, indicating once again that managers were unable to identify their specific exposure to pandemic risks.

Notice that the Fama–French (1997) industries of food and candy-soda had high counts of pandemic-related words and some of the best relative stock performance (although still negative) of any industry. Only the firearms industry had higher stock returns than the food and candy-soda industries. Between February 3, 2020, and March 23, 2020, the stock prices of firms in the oil industry plummeted by nearly 60% as the entire economy shut down and Saudi Arabia and Russia engaged in an all-out oil price war.^8^ Oil had the lowest stock returns of any Fama–French (1997) industry. Nonetheless, the oil industry had one of the lowest pandemic-related word counts (less than 6% oil companies using one of our target words). In hindsight, oil executives should have warned their shareholders about the industry’s enormous downside risk in the event of a pandemic.

## Conclusion

By means of the periodic disclosures mandated by federal securities law, managers are obligated to inform existing and prospective shareholders of any reasonable risks that could affect the value of their shares. Although we know that some firms were positively affected by the COVID-19 pandemic (e.g., Amazon, Clorox, Netflix, and Zoom Video Communications), the stock price of many others declined significantly. We find a slight positive correlation between industry performance and the use of pandemic-related words, as opposed to the expected negative correlation.

Managers with a thorough understanding of their operations should have anticipated the indirect effects of a pandemic on specific industries, such as supply chain disruptions. When assessing the pandemic-related risk factors facing a company’s operations, merely mentioning a general economic catastrophe does not appear to offer shareholders sufficient insight. Perhaps with hindsight, we would have anticipated that more than 21% of firms would disclose pandemic risk as a potential and significant business disruption.

## Data Availability

Our datasets will be posted in a publicly available repository after publication.

## Appendix: Parsing and data methodology

Below we provide a brief outline of how the sample was created.

- https://www.sec.gov/Archives/ contains, by year and quarter, a master.idx file that is a pipe-delimited listing of all SEC filings.
- Using the four master.idx files for quarters 1–4 in 2018 which contain file pointers for each filing, download all 10-K type filings, i.e., “Form Type” = [‘10-K’, ‘10-K405’, ‘10KSB’, ‘10-KSB’, ‘10KSB40’]
- Use the EDGAR header in the filing to identify the SIC classification and convert this into a Fama–French (1997) industry classification.
- After deleting all XBRL, HTML, and ASCII-encoded segments with a Python program, we use a series of regular expressions to extract the section labeled “ITEM 1A: RISK FACTORS”.
- From the remaining text, use a regular expression to identify and tabulate the target phrases:
  TARGET_LIST = [“PANDEMIC”, “EPIDEMIC”, “CORONA\s?VIRUS”, “CONTAGIOUS\sDISEASE”, “CONTAGIOUS\sILLNESS”,
  “INFECTIOUS\sDISEASE”, “INFECTIOUS\sOUTBREAK”, “FEAR\sOF\sCONTAGION”, “INFLUENZA\sVIRUS”, “AVIAN\sFLU”, “H1N1”, “SWINE\sFLU”, “\sMERS\s”, “\sEBOLA\s”, “\SARS\s”, “LOCALIZED\sILLNESSES”, “HEALTH\sCONCERNS”, “OUTBREAK\sOF\sDISEASE”]

## Abbreviations

SEC: Securities and Exchange Commission
EDGAR: Electronic Data Gathering, Analysis, and Retrieval
MD&A: Management’s Discussion and Analysis
CDC: Centers for Disease Control and Prevention

## Footnotes

1 There are variants of the 10-K, including quarterly filings (10-Qs). We focus only on the annual 10-K, 10-K405, 10KSB, 10-KSB, and 10KSB40 filings and do not include amended filings (like 10-K/A or 10-K405/A) in the sample.

2 Other published papers that analyze the link between COVID-19 and financial markets include Gormsen and Koijen (2020), Liu et al. (2020), Salisu and Vo (2020), Cheema-Fox et al. (2021), Demers et al. (2021), Hong, Bian, and Lee (2021), Mazur et al. (2021), Salisu and Obiora (2021), and Song, Yeon, and Lee (2021). Fernandes (2020) and Ozili and Arun (2022) discuss the impact of COVID on the broader global economy.

3 See, for example, Verrecchia (1983), Dye (1985), or Guttman et al. (2014). Useful reviews appear in Verrecchia (2001) and Leuz and Wysocki (2016).

4 See, for example, Bill Gates’ April 3, 2015 TED Talk “The next outbreak? We’re not ready”; “Bill Gates Warns of Epidemic That Could Kill over 30 Million,” Forbes, 2/19/2017; “The World is Not Ready for the Next Pandemic,” *Time*, 5/4/2017; and “Is America Ready for a Global Pandemic,” *The Atlantic*, 7/2018.

5 See https://www.cdc.gov/h1n1flu/estimates_2009_h1n1.htm.

6 Other survey and empirical papers in the literature which use different techniques include Kou et al. (2014), Wen et al. (2019), Kou et al. (2021), and Li et al. (2021).

7 Note that for terms including spaces, like *contagious disease*, we count any collection of characters where any form of whitespace occurs between the two words, including no whitespace (e.g., we would count the case where a line feed occurs between *contagious* and *disease*). Also, we count *corona virus* and *coronavirus* as the same token.

8 As noted in a March 8, 2020 CNBC article, “Experts are now calling dramatically lower crude prices as major OPEC and non-OPEC producers ready for an all-out price war after failing to reach an output cut agreement Friday, in a sudden U-turn from previous attempts to support the oil market as the new coronavirus hammers global demand.” See https://www.cnbc.com/2020/03/08/opec-deal-collapse-sparks-price-war-20-oil-in-2020-is-coming.html.
